# Measuring Gray Matter and White Matter Damage in MS: Why This is Not Enough

**DOI:** 10.3389/fneur.2015.00056

**Published:** 2015-03-17

**Authors:** Christian Enzinger, Franz Fazekas

**Affiliations:** ^1^Department of Neurology, Medical University of Graz, Graz, Austria; ^2^Division of Neuroradiology, Department of Radiology, Medical University of Graz, Graz, Austria

**Keywords:** multiple sclerosis, plasticity, adaptation, brain, function, gray matter, white matter, MRI

## Introduction

Over the past years, progress in cerebral magnetic resonance imaging (MRI) technology has increased the possibilities to quantify MS-related tissue changes, starting from lesion assessment in the white matter (WM) to the quantification of microstructural changes of the whole brain (1). This was expected to give full insight into the causes of MS patients’ deficits but despite all achievements, the current plethora of MRI metrics still provides no complete explanation for the clinical condition on a group (2) and even less so on an individual level. However, within the scope of personalized medicine, this remains an important goal to better understand and ultimately minimize the functional impact of MS-related tissue damage.

In this context, functional cerebral changes including adaptation and plasticity are strong contributors to the apparent clinical consequences of MS and are likely to explain part of the “morphological-clinical gap” (3), notwithstanding ongoing controversies what patient deficits to consider and how to assess them. Against this background, we critically review the development and current state of techniques to assess gross MS-related morphologic damage and their contribution to understand the clinical consequences of MS (disability, and also cognitive problems and fatigue) and the obvious modulating roles of cerebral adaptation and plasticity as unraveled by functional MRI (fMRI). From existing data, we suggest that it is unlikely to ever achieve a satisfactory level of explanation and prediction of an individual patient’s condition-based solely on morphologic information, although such insights might be better suited to define disease progression than clinical assessment.

## White Matter Damage in MS

MS has traditionally been viewed as multifocal WM disease, and depicting lesions disseminated throughout the CNS using conventional MRI has become indispensable in early diagnosis and management (4). However, T2-weighted MRI lacks pathological specificity (5).

### Focal white matter pathology

While the basic features of MS pathology constitute inflammation, demyelination in WM and GM, and diffuse neurodegeneration within the entire CNS, the individual components of the pathological spectrum vary quantitatively between early relapsing and late progressive MS (6). Moreover, remyelination of existing lesions may be extensive in a subset of patients and fail in others (6). All these components cannot be sufficiently assessed on T2-weighted MRI.

Compared to T2-hyperintense lesions, so-called “black holes” (severely and persistent hypointense lesions on T1-weighted MRI) have been shown to offer a more specific marker of matrix destruction and axonal loss (7). However, their definition is variable and strongly dependent on scanning parameters, which prohibits closer quantitation.

### Non-conventional MRI to quantify lesional white matter damage

A more refined insight into the composition of lesions may be gained by non-conventional techniques like magnetization transfer (MT) imaging (MTI), diffusion-weighted imaging (DWI), and diffusion tensor imaging (DTI).

MT-ratio (MTR) changes may precede the formation of active MS lesions by months, indicating changes in the macromolecular composition of pre-lesional WM long before a lesion becomes visible on conventional MRI (8). Once a lesion has formed, i.e., becomes apparent on T2-weighted MRI, MTI may serve to follow the evolution and fate of affected parenchyma. Comparison with histopathology has shown good correlations with both demyelination and remyelination, and also just with fiber or neuronal density (5, 9). In a longitudinal trial, MTR-changes followed different temporal evolutions and were ongoing in different lesion regions for at least 3 years after lesion formation (10).

Diffusion-weighted imaging and DTI yield different insights. Diffusion measures the microscopic Brownian motion of water molecules, which is hindered by cellular structures (e.g., cell membranes and axonal cytoskeletons). In general, low-fractional anisotropy (FA) and high-mean diffusivity (MD) are found in MS lesions, but values are highly heterogeneous. Unfortunately, few studies investigated pathological correlates of DWI in MS. Surprisingly, at post-mortem, MD and FA correlated more strongly with myelin content than with axonal count and gliosis in one study (11).

### Non-conventional MRI to quantify diffuse white matter damage

Diffusion-weighted imaging/DTI and MTI played important roles in shaping the notion that MS not only consists of focal T2-lesions but also affects WM in a more widespread manner. Thus, normal appearing white matter (NAWM) in MS, in fact, is not normal, but demonstrates subtle microstructural abnormalities outside lesions. Histogram analyses have frequently been used to explore respective MRI metrics across the whole brain (12–14). Using MTI, MS patients consistently showed both lower average MTR and lower peak height (15) and respective DTI analyses demonstrated higher average MD, lower histogram peak height MD, and lower average FA (14, 16) compared to healthy controls, respectively. Lower FA values close to and higher values far from MS lesions suggest Wallerian degeneration (17), but MD and FA of NAWM only partially correlate with the extent and severity of focal lesions, indicating other factors like astrocytic hyperplasia, patchy edema, perivascular infiltration, demyelination, and axonal loss also contribute to such abnormalities (14).

### Strategic location of white matter damage

Assessing the strategic location of WM damage using lesion probability maps (LPM) or diffusion-based tractography (DBT) represents another promising approach to improve upon morphological-clinical correlations. Thus, LPM in different MS phenotypes revealed associations between specific neurologic and cognitive deficits with lesion accumulation in distinct, anatomically plausible, regions, but only to limited extent (18). Using tract-based spatial statistics [TBSS; a fully automated, whole-brain diffusion analysis method (14)] to identify loci where reduced WM-tract FA predicted impaired cognitive performance in MS patients, cognitively relevant tract localizations only partially overlapped with areas of high-lesion probability, but identified tract localizations were found to interconnect cortical regions involved in cognitive processing (19). Thus, there is evidence that abnormalities in strategic brain WM tracts contribute to cognitive impairment in MS, but the identified regions vary between studies and it is unlikely that such analyses could be performed on an individual level.

## Gray Matter Damage in MS

### Gray matter pathology

Autopsy studies have demonstrated that MS is also associated with focal lesions in and diffuse demyelination of GM (20, 21). The depiction of these kinds of MS-related damage has been and still is a challenge for MRI.

### Focal gray matter damage

Intrinsically, low-myelin densities in the cortex (where demyelination generates little contrast), the often small size of lesions, and partial volume effects of cerebrospinal fluid impede the detection of cortical GM lesions on conventional MRI (21). Introduction of the double-inversion recovery (DIR) sequence with superior sensitivity compared to T2- and fluid-attenuated-inversion-recovery (FLAIR) sequences improved this situation, but post-mortem comparison showed that 80% of lesions still remain undetected (20, 21). Interestingly, MRI-visible cortical lesions do not differ from MRI-invisible lesions in their pathological profiles (20). Combination with other sequences such as phase-sensitive inversion recovery and T1-weighted 3D-spoiled gradient-recalled echo and ultra-high-field scanners might help to partly overcome these problems (20, 22), but this approach is not realizable in clinical settings. Importantly, despite representing the commonest lesion type, subpial cortical lesions largely escape detection by MRI (20). Deep GM pathology is somewhat easier to depict than cortical GM pathology as lesions in deep GM structures, spinal cord, and hippocampus are generally mixed GM/WM lesions and slightly more inflammatory (21).

### Diffuse gray matter damage

Cortical thickness is reduced in MS (23) and a mean cortical thinning of 10% has been found independently of cortical lesions, suggesting mechanisms besides cortical demyelination contribute to cortical atrophy (21). A recent study combining post-mortem imaging and histopathology to explore the underpinnings of cortical atrophy identified neuronal density, neuronal size, and axonal density as predictors of cortical GM volume in long-standing MS (24). GM constitutes about 65% of brain parenchymal tissue, and atrophy of GM largely drives whole-brain atrophy in MS (20). GM atrophy in MS occurs both at global and regional level and can be quantitated using MRI. It also does not directly correlate with the number of WM lesions and diffuse NAWM damage, suggesting partially independent pathological processes (22). Unfortunately, GM volume measures are inherently non-specific and reveal little about the exact cause of tissue injury (20).

### Non-conventional MRI to quantify gray matter damage

Analogous to but less frequent than respective WM studies, DWI/DTI and MTI have also been used to quantify GM damage in MS. Consistent with differences in pathology, DTI detected higher MD and FA in cortical lesions than in WM lesions of MS patients (22). New approaches to study cortical MTR changes include segmentation of the cortex to obtain separate information on outer and inner bands, where the lowest outer cortical MTR was seen in secondary progressive MS, consistent with post-mortem findings of more extensive subpial pathology in this group (25).

### Strategic location of gray matter damage

Extensive GM involvement has been associated with cognitive decline, motor deficits, fatigue, painful syndromes, and ocular motility disturbances in MS. In this respect, the thalamus has been highlighted, as it relays sensory information to higher cortical centers that influence cognition (26). A strategic significance has also been demonstrated for the hippocampus, as lesions in this area strongly correlate with impaired visuospatial memory and processing speed (20, 21). Further, using LPMs of DIR images, cortical MS lesions have been demonstrated to be mainly distributed in the frontal and temporal lobes, with prominent involvement of motor and anterior cingulate cortices (27).

## Contribution of White and Gray Matter Damage to Explaining Clinical Deficits

All the above techniques have greatly enhanced our understanding of the complexity of MS tissue damage and provide possibilities to assess the structural changes associated with MS in ever more detail. At group level, several discussed metrics alone or in combination have shown good correlation with measures of disability or cognition [e.g., Ref. (28)] and even simple MRI markers appear to be an excellent surrogate for treatment response (29). However, these complex insights have not transformed into a sufficient capacity to explain or even predict a patient’s functioning and clinical deficits, and are far from having reached utility in daily clinical practice. We suggest that the reasons for this are not only the complexity of MS-related damage – which is certainly even much greater than addressed above (e.g., consider the role of other CNS compartments such as the spinal cord) – but rather the individual variability in processes of plasticity and adaptation.

## Functional MRI as One Approach to Gain Functional Information

Functional MRI studies of visual, cognitive, and motor systems consistently demonstrated functional changes in all MS phenotypes, characterized by altered activation of regions normally devoted to performance of a task or recruitment of additional areas compared to healthy subjects (3, 22, 30, 31). Given the correlation between functional and structural abnormalities (30), the former appear to partly limit the functional consequences of structural damage in MS. fMRI abnormalities already occur in CIS, but differences in activation patterns between phenotypes are striking (32, 33), suggesting profound changes in the functional organization of the MS brain with disease progression. Final exhaustion of adaptive capacity may constitute one key factor for unfavorable clinical evolution or cognitive decline, although the decisive factors driving transition from adaption to maladaptation are unknown. However, maladaptation in MS is not only the consequence of the final exhaustion of adaptive plasticity but it may also be expressed in early stages of the disease as enhanced functional connectivity (34). While patients with “long-term low disability MS” may functionally withstand considerable amounts of brain tissue damage, others already suffer from severe disability (35). Individual differences in brain reserve and cognitive reserve thereby might play a role (36–38). Combining MRI measures of structural damage with those of abnormal functional and structural connectivity (3) using resting state fMRI (13, 39) appears promising to further elucidate such relationships at group level, but this would necessitate prospective longitudinal studies (40) with long-term follow-up, ideally in multi-center settings (41).

## Conclusion and Future Directions

Despite increasing level of detail, morphological insights using MRI by nature only allow assessment of (micro)structural disease-related processes in MS-brains. fMRI enabled detection of paralleling adaptive cerebral changes, which – besides the stage and dynamics of the disease – most likely are also modulated by individual differences (“functional reserve”). Interpretations of such changes are corroborated and usefully augmented by concomitant assessment of morphological MRI changes. Yet, measurement of brain damage by structural MRI alone clearly does not suffice to comprehensively appreciate the consequences of the disease, although it is ideal for specific questions (e.g., assessment of disease activity, remyelination, or evolution of atrophy). To better understand mechanisms of functional adaption in MS, longitudinal studies including (micro)structural and functional MRI in large registries of early MS followed for many years are needed. Whether this will finally allow judging capacity for adaption at the individual level remains unclear.

## Conflict of Interest Statement

This opinion article was drafted in the absence of any commercial or financial relationships that could be construed as a potential conflict of interest. Declaration of financial relationships and other activities: Dr. Christian Enzinger has received funding for travel and speaker honoraria from Biogen Idec, Bayer Schering Pharma, Merck Serono, Novartis Genzyme, and Teva Pharmaceutical Industries Ltd./sanofi-aventis; research support from Merck Serono, Biogen Idec., and Teva Pharmaceutical Industries Ltd./sanofi-aventis; serving on scientific advisory boards for Bayer Schering Pharma, Biogen Idec, Genzyme, Merck Serono, Novartis, and Teva Pharmaceutical Industries Ltd./sanofi-aventis; academic editor for PLOSOne. Dr. Franz Fazekas serves on scientific advisory boards for Bayer-Schering, Biogen Idec, Genzyme, Merck Serono, Pfizer, Novartis, and Teva Pharmaceutical Industries Ltd.; serves on the editorial boards of Cerebrovascular Diseases, Multiple Sclerosis, the Polish Journal of Neurology and Neurosurgery, Stroke, and the Swiss Archives of Neurology and Psychiatry; and has received speaker honoraria and support from Biogen Idec, Bayer Schering, Merck Serono, Novartis, Pfizer, Sanofi-Aventis, Shire and Teva Pharmaceutical Industries Ltd.
